# Physical Interaction between Embryonic Stem Cell-Expressed Ras (ERas) and Arginase-1 in Quiescent Hepatic Stellate Cells

**DOI:** 10.3390/cells11030508

**Published:** 2022-02-01

**Authors:** Silke Pudewell, Jana Lissy, Hossein Nakhaeizadeh, Mohamed S. Taha, Mohammad Akbarzadeh, Soheila Rezaei Adariani, Saeideh Nakhaei-Rad, Junjie Li, Claus Kordes, Dieter Häussinger, Roland P. Piekorz, Miriam M. Cortese-Krott, Mohammad Reza Ahmadian

**Affiliations:** 1 Institute of Biochemistry and Molecular Biology II, Medical Faculty and University Hospital Düsseldorf, Heinrich Heine University Düsseldorf, 40225 Düsseldorf, Germany; silke.pudewell@hhu.de (S.P.); Lissy.jana@gmail.com (J.L.); hossein.nakhaeizadeh@gmail.com (H.N.); ms_taha@yahoo.com (M.S.T.); mohammad.akbarzadeh@mpi-dortmund.mpg.de (M.A.); soheila.rezaei@mpi-dortmund.mpg.de (S.R.A.); s.nakhaeirad@um.ac.ir (S.N.-R.); Roland.Piekorz@hhu.de (R.P.P.); 2 Research on Children with Special Needs Department, Medical Research and Clinical Studies Institute, National Research Centre, 12622 Dokki, Cairo, Egypt; 3 Department of Chemical Biology, Max-Planck Institute of Molecular Physiology, Otto-Hahn-Strasse 11, 44227 Dortmund, Germany; 4 Stem Cell Biology and Regenerative Medicine Research Group, Institute of Biotechnology, Ferdowsi University of Mashhad, Mashhad 9177948974, Iran; 5 Myocardial Infarction Research Laboratory, Department of Cardiology, Pulmonology, and Vascular Medicine, Medical Faculty and University Hospital Düsseldorf, Heinrich Heine University Düsseldorf, 40225 Düsseldorf, Germany; Junjie.Li@hhu.de (J.L.); miriam.cortese-krott@med.uni-duesseldorf.de (M.M.C.-K.); 6 Clinic of Gastroenterology, Hepatology and Infectious Diseases, Medical Faculty of the Heinrich-Heine University, 40225 Düsseldorf, Germany; Claus.Kordes@hhu.de (C.K.); haeussin@hhu.de (D.H.)

**Keywords:** arginase 1, ARG1, embryonic stem cell-expressed Ras, ERas, hepatic stellate cells, quiescence, iNOS, L-arginine, L-ornithine, polyamines, spermidine, spermine, urea cycle

## Abstract

Embryonic stem cell-expressed Ras (ERas) is an atypical constitutively active member of the Ras family and controls distinct signaling pathways, which are critical, for instance, for the maintenance of quiescent hepatic stellate cells (HSCs). Unlike classical Ras paralogs, ERas has a unique N-terminal extension (Nex) with as yet unknown function. In this study, we employed affinity pull-down and quantitative liquid chromatography-tandem mass spectrometry (LC–MS/MS) analyses and identified 76 novel binding proteins for human and rat ERas Nex peptides, localized in different subcellular compartments and involved in various cellular processes. One of the identified Nex-binding proteins is the nonmitochondrial, cytosolic arginase 1 (ARG1), a key enzyme of the urea cycle and involved in the de novo synthesis of polyamines, such as spermidine and spermine. Here, we show, for the first time, a high-affinity interaction between ERas Nex and purified ARG1 as well as their subcellular colocalization. The inhibition of ARG1 activity strikingly accelerates the activation of HSCs ex vivo, suggesting a central role of ARG1 activity in the maintenance of HSC quiescence.

## 1. Introduction

Embryonic stem cell expressed Ras (ERas) is a unique member of the Ras superfamily that was first identified in undifferentiated mouse embryonic stem cells (ESCs). ERas expression has also been reported in gastric cancer, breast cancer, and neuroblastoma cell lines [1,2,3,4,5,6] and has been proposed to be critical for growth and tumor-like properties in these cells [1,7]. We have already demonstrated that ERas is expressed in quiescent rat hepatic stellate cells (HSCs), where it controls HSC quiescence in the liver through distinct signaling pathways, including PI3K-AKT and MST-LATS-YAP [8]. HSCs are pericytes that reside in close contact with sinusoidal endothelial cells in the space of Disse [9]. This unique space in liver sinusoids is bordered by endothelial cells and hepatocytes providing a niche that helps to sustain HSC quiescence [10]. Once activated, HSCs show typical characteristics and functions of mesenchymal stem cells and have therefore been classified as such [11]. A mechanism that has since been described for pericytes of other organ systems as well [12]. An intact stem cell niche is crucial for the maintenance of stemness, differentiation and developmental fate decisions of stem cells [10,13]. In a normal, healthy liver, HSCs represent 5–8% of the total liver cells and store about 85% of the body’s vitamin A as retinyl palmitate in membrane-coated vesicles [9]. The expression of neural and mesodermal markers, i.e., glial fibrillary protein (GFAP) and desmin, are known to be displayed in a quiescent phenotype. Following liver injury, quiescent HSCs (qHSCs) activate and develop into proliferative and contractile myofibroblast-like cells (aHSCs), revealing profibrogenic transcriptional properties and accounting for extracellular matrix accumulation [14,15]. Activated HSCs show downregulation of GFAP and ERas, upregulation of α-smooth muscle actin (α-SMA) and collagen type I, as well as loss of lipid droplets [8,10].

Arginase (EC 3.5.3.1.) is a manganese-containing enzyme that catalyzes the hydrolysis of L-arginine to L-ornithine and urea. It is a key enzyme of the hepatic urea cycle but is also expressed in extrahepatic tissues lacking a complete urea cycle. There are two paralogs that differ in expression, regulation, and localization [16]. The nonmitochondrial, cytosolic enzyme, arginase 1 (ARG1), is the predominant paralog in the liver and red blood cells [17], whereas the mitochondrial arginase 2 is mainly expressed in extrahepatic tissues [16,18,19,20]. L-arginine is not only a substrate for arginase, but can alternatively be converted to nitric oxide (NO) and L-citrulline by nitric oxide synthases (NOS). Thus, one biological function of arginase in extra-hepatic organs lies in the regulation of NO synthesis, by competing with NOS for the common substrate L-arginine [21] and also participates in numerous inflammatory diseases by the downregulation of NOS activity, the induction of fibrosis and tissue regeneration [22]. Interestingly, the much higher K_m_ of the NO synthases (2–20 µM) [23] and the quite low V_max_ (1 µmol/min/mg) [23] in contrast to the low K_m_ (2–20 mM) [24] and high V_max_ (1400 µmol/min/mg) [25] of arginase together with a high extracellular and low intracellular L-arginine concentration, respectively, describes the arginine paradox, which might be solved by the consumption of different pools of L-arginine by these two competing enzymes.

The aim of this study was to identify the ERas interaction partners that play a role in the homeostasis of HSC quiescence. Using a proteomic approach, we identified numerous novel potential ERas Nex interactors, including ARG1, that are involved in diverse cellular processes. We characterized ERas-ARG1 interaction on protein and cellular levels. Furthermore, we demonstrate the L-arginine metabolism as a central mediator of HSC quiescence and propose that the ARG1-polyamine axis plays a role in hepatic stellate cell homeostasis.

## 2. Materials and Methods

### 2.1. Reagents

Dulbecco’s modified Eagle medium (DMEM), fetal bovine serum (FBS) and penicillin/streptomycin were obtained from Gibco^®^ Life Technologies. Primary and secondary antibodies for immunoblotting and immunocytochemistry are listed in the Supplementary Table S2. The nucleotides mant-GppNHp and Gpp(CH_2_)p were obtained from Jena Bioscience GmbH. CellTiter-Blue^®^ was purchased from Promega (Mannheim, Germany).

### 2.2. Constructs and Proteins

ARG1 (P05089; aa 1–322), human ERas FL (Q7Z444; aa 1–233), ΔC (aa1-201), Nex (aa 1–38), rat ERas FL (D3ZTE4; aa 1-227), Nex (aa 1–38), and ΔC (aa 1–201) were cloned into pMal-c5X-His or pGEX-4T1-N-TEV. Expression was carried out in *Escherichia coli*. Proteins and peptides were prepared using glutathione and Ni-NTA-based affinity and size exclusion chromatography as described previously [26]. 

### 2.3. Cell Culture 

HSC isolation was done as described previously [8]. HSCs were seeded and cultured for 4 or 8 days in DMEM supplemented with 10% FBS and 1% penicillin/streptomycin. HEK293 were cultured in DMEM supplemented with 10% FBS and 1% penicillin/streptomycin. The cells were cultured in an exponential growth phase at 37 °C, 5% CO_2_, and 95% humidity. Transfection was performed by TurboFect™ Transfection Reagent (Thermo Fisher Scientific, Waltham, MA, USA) following the manufactures’ protocol. 

### 2.4. Affinity Pull-Down Assay

GST-fusion proteins were immobilized on GSH agarose beads and subsequently mixed with purified proteins or total cell lysates and incubated for 1 h, at 4 °C to pull down associating proteins. The beads were washed four times, boiled in SDS (sodium dodecyl sulfate) loading buffer at 95 °C for 5 min. Samples were separated using SDS polyacrylamide gels. The gels were either immunoblotted and stained with specific antibodies as described previously [8] or directly stained with Coomassie brilliant blue (CBB). 

### 2.5. Immunoprecipitation

Immunoprecipitation was performed as described previously [8]. In brief, freshly isolated HSC cells or HEK293 cells were lysed in immunoprecipitation buffer (20 mM Tris-HCl, pH 7.4, 150 mM NaCl, 5 mM MgCl_2_, 0.5% Nonidet P-40, 10 mM β-glycerolphosphate, 0.5 mM Na_3_VO_4_, 10% glycerol, EDTA-free protease inhibitor). Total cell lysates of HSCs were incubated with ERas antibody or IgG control respectively for 1 h, at 4 °C, followed by 1 h incubation at 4 °C with Protein G beads. TCL of HEK293 cells was incubated with GFP-coupled nanobeads (kindly provided by Manuel Franke) for 1 h, at 4 °C as described before [8]. Eluted proteins were finally denatured in SDS loading buffer at 95 °C and analyzed by immunoblotting as described previously [8].

### 2.6. Quantitative Real Time-Polymerase Chain Reaction

Cells were disrupted by QIAzol lysis reagent (Qiagen, Hilden, Germany) and total RNA was extracted via RNeasy plus kit (Qiagen) according to the manufacturer’s protocol. The quality and quantity of isolated RNA samples were analyzed on 1% agarose gels and using a Nanodrop spectrophotometer (Thermo Fisher Scientific), respectively. Possible genomic DNA contaminations were removed using the DNA-free™ DNA Removal Kit (Ambion, Life Technologies, Carlsbad, CA, USA). DNase-treated RNA was transcribed into complementary DNA (cDNA) using the ImProm-II™ reverse transcription system (Promega, Madison, WI, USA). Quantitative real-time reverse transcriptase-polymerase chain reaction (qRT-PCR) was performed using SYBR Green reagent (Life Technologies). Primer sequences are listed in Supplementary Table S3. The 2^−∆Ct^ method was employed for estimating the relative mRNA expression levels. *Hprt1* was used as a housekeeping gene.

### 2.7. Arginase Activity Assay 

Purified ARG1 from *E. coli* was activated by incubation with 10 mM MnCl_2_, 50 mM Tris-HCl pH 7.5, at 55 °C for 10 min. ARG1 activity was determined by mixing 100 nM of ARG1 with increasing concentrations of L-arginine (250–2000 µM) at 37 °C. Samples were taken at various time points between 20 s and 5 min and denatured at 95 °C for 5 min. The L-arginine concentration was determined via HPLC (Beckman Coulter System Gold, LC118/LC116) in a reversed-phase Discovery C18 column (250 mm). As a mobile phase, 10% acetonitrile and 20 mM Na_2_HPO_4_ monohydrate with a final pH of 6 was used. The absorbance was measured at 210 nm with a flow rate of 1 mL/min at room temperature. Michaelis–Menten kinetics were determined by plotting the reaction velocity (v) as a function of the L-arginine concentration using Grafit 5.0.13.

Higher concentrations of urea were determined by a colorimetric urea assay [27]. Cell lysates were mixed with increasing concentrations of L-arginine (1–50 mM) at incubated at 37 °C. Samples were taken at various time points and the reaction was stopped by adding 400 µL acidic mixture consisting of H_2_SO_4_, H_3_PO_4_ and H_2_O (1:3:7). In cell culture supernatants, urea production was quantified by mixing 50 µL of the medium with 400 µL acidic mixture. Urea concentration was quantified by the addition of 25 µL 9% isonitrosopropiophenone (dissolved in 100% EtOH) and incubation for 45 min at 100 °C. The reaction was kept in the dark for 10 min at room temperature before measuring the absorbance at 540 nm in a TECAN Infinite M200 PRO reader. A urea standard was used to calculate exact concentrations.

### 2.8. Synthetic Liposomes and Liposome Sedimentation 

Synthetic liposomes were generated by mixing and sonicating 500 µg total lipids: 20% (*w*/*w*) phosphatidylethanolamine (PE), 45% (*w*/*w*) Phosphatidylcholine (PC), 20% (*w*/*w*) Phosphatidylserine (PS), 10% (*w*/*w*) cholesterol, and 5% (*w*/*w*) phosphatidylinositol 4, 5-bisphosphate (PIP_2_). Lipids were dried out using light nitrogen stream and obtained lipid film was hydrated in 300 µL buffer containing 20 mM HEPES (pH 7,5), 50 mM NaCl, 5 mM MgCl_2_, and 3 mM DTT. The lipid suspension was sonicated once at low settings and extruded 21 times through a 0.2 µm pore size membrane filter. 

For the liposome sedimentation assay, liposomes were mixed with an excess amount of purified ARG1 protein and incubated 30 min at 4 °C on a rotor. The mixture was centrifuged for 30 min at 20.000× *g* and 4 °C. The supernatant (which contains unbound proteins) was mixed with 5× SDS (20%) loading buffer and the liposome pellet (containing liposome bound proteins) were resuspended in an equal amount of 1× SDS loading buffer. The samples were analyzed via SDS gel electrophoresis and Coomassie staining or immunoblotting as described before [8].

### 2.9. Mass Spectroscopy and Data Analysis of ERas Nex-Binding Proteins

For mass spectrometric analysis of ERas Nex binding proteins, SDS gel fragments were cut from each lane of the affinity pull-down assay. The gel pieces were reduced, alkylated, and digested by trypsin. The resulting digest mixtures were analyzed by mass spectrometry as described in [28]. Peptides extracted with 0.1% trifluoroacetic acid were subjected to a liquid chromatography system (RSLC, Dionex/Thermo Scientific, Idstein, Germany) equipped with an Acclaim PepMap 100 C18 column (75 µm inner diameter, 50 cm length, 2 mm particle size from Dionex/Thermo Scientific, Idstein, Germany) coupled to an Orbitrap Elite mass spectrometer (Thermo Scientific, Bremen, Germany) essentially as described in [28]. For protein and peptide identification and quantification, raw files were further processed using the MaxQuant software suite version 1.3.0.5 (Max Planck Institute of Biochemistry, Planegg, Germany). Database searches were carried out against the UniProt database (release 06.2013) using standard parameters. Label-free quantification was done using the “match between runs” option with a time slot of 2 min. Peptides and proteins were accepted at a false discovery rate of 1% and proteins with quantitative information available for at least three analyzed samples were subjected to subsequent statistical analysis. Protein quantification was performed using the SAM algorithm [29] implemented in Perseus version 1.2.7.4 (Max Planck Institute of Biochemistry, Planegg, Germany) on log-transformed data (false discovery rate threshold: 0.01). Missing values were replaced by imputation (width: 0.3; downshift: 1.8).

### 2.10. Gene Ontology Analysis

Gene Ontology (GO) terms for the biological processes, molecular function, and cellular location of ERas Nex interacting proteins, including isoforms, paralogs, or related proteins were achieved using the PANTHER database [30].

### 2.11. Surface Plasmon Resonance (SPR)

For kinetic analysis of the interaction between ERas and ARG1, a Biacore X100 system was used together with CM5 sensor chips (GE Healthcare Life Sciences, Uppsala, Sweden). An anti-GST antibody (Supplementary Table S2) was immobilized to the dextran surface of a CM5 sensor chip using the GST capture kit (GE Healthcare Life Sciences). Afterwards, 10 µM of purified GST-*hs*Nex was introduced to the immobilized GST-antibody at 25 °C (30 µL/min). Increasing concentrations of MBP-ARG1 (contact time: 90 sec, 30 µL/min) were injected in a multicycle mode and dissociation was measured at the end of the injection of the final concentration for a period of 300 sec. The dissociation constants (K_d_) were calculated using BIAevaluation (version 2.0.1) by the Langmuir 1:1 model and the GraFit 5 version. All SPR measurements were carried out at 25 °C in a buffer, containing 10 mM HEPES, pH 7.4, 150 mM NaCl, and 0.05 % (*v*/*v*) surfactant P20 (GE Healthcare Life Sciences, Uppsala, Sweden).

### 2.12. Detergent-Free Subcellular Fractionation of HSCs

Subcellular fractionation of HSCs (d0) was conducted by using a differential centrifugation method combined with detergent-free buffers and sucrose cushions as described previously [31].

### 2.13. Confocal Imaging

Confocal images were obtained using a LSM 880-microscope (Zeiss, Jena, Germany). Immunostaining was performed as described previously [32]. Primary antibodies and secondary antibodies are listed in the Supplementary Table S2. 

### 2.14. Cell Viability Assay

To determine cell viability which directly correlates to cell number, 5000 cells/well were seeded in 96-well plates in 100 µL medium. On the desired day, 20 µL of CellTiter-Blue solution was added to each well and the fluorescence was determined at 590 nm using a TECAN Infinite M200 PRO reader. The cells were incubated under normal growth conditions and the fluorescence was measured again after one and two hours. The fluorescence was plotted against the time and the slope determined the relative number of viable cells. 

### 2.15. Oil Red O Staining

For the ORO staining, HSCs were cultured in 24-well plates and washed 3 times with phosphate-buffered saline (PBS). Afterwards, the cells were fixed with 4% PFA for 10 min at room temperature, followed by a 20 min incubation with the 1x ORO working solution as described [33,34]. The cells were rinsed with 60% isopropanol and imaged with a bright field microscope. 

### 2.16. Statistical Analysis

The data were evaluated using GraphPad Prism 6 software. For variance analysis, an ordinary one-way or two-way analysis of variance test was performed using Turkey’s or Dunnett’s multiple comparison test or paired or unpaired *t*-test as indicated. Results were considered significant with *p* < 0.05 (* *p* < 0.05, ** *p* < 0.01, *** *p* < 0.001, **** *p* < 0.0001).

## 3. Results

### 3.1. Novel Binding Partners of ERas Are Involved in Multiple Cellular Processes

**Figure 1:**
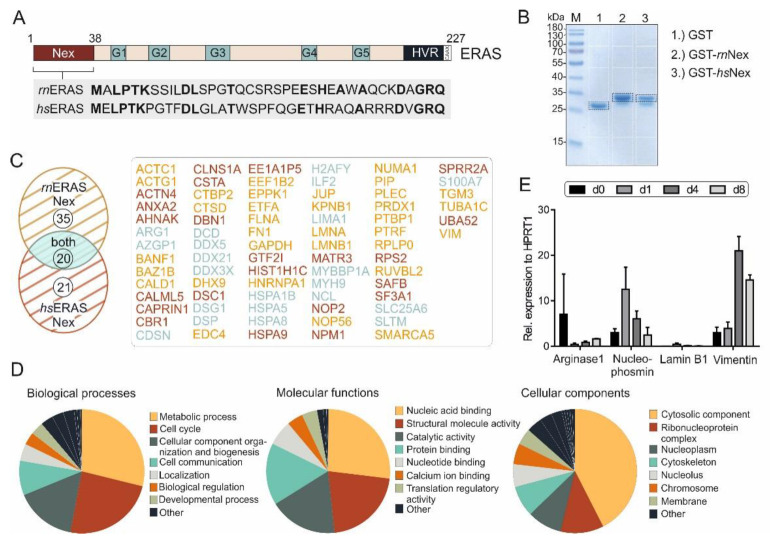
ERas N-terminal extension and its novel binding partners. (**A**) ERas contains, in addition to five motifs (G1-5) in its G domain, an N-terminal extension (Nex) and a C-terminal hypervariable region (HVR) ending with a consensus sequence known as CAAX. An alignment of ERas N-terminus of *Rattus norvegicus* (*rn*) and *Homo sapiens* (*hs*) shows a sequence identity (bold amino acids) of 42%. (**B**) Purified GST, GST-*rn*Nex or GST-*hs*Nex proteins were used for affinity pull-down experiments with GSH beads in total cell lysates, to identify ERas Nex binding partners. Bound proteins were resolved on a 10% SDS gel and stained with Coomassie brilliant blue. White boxes indicate different gel fragments excised for mass spectrometric (MS) analysis. Black boxes indicate GST, GST-*rn*Nex or GST-*hs*Nex and were excluded from MS analysis (n = 3). (**C**). Evaluation of MS analysis revealed in total 76 ERas Nex binding partners from which 35 preferentially interact with *rn*Nex (yellow), 21 with *hs*Nex (red) and 20 with both (blue) (for more detail see Table S1). (**D**) Gene Ontology analysis of identified ERas Nex interacting proteins categorized according to biological processes, molecular functions, and subcellular localizations. Biological processes (left panel) were predominantly classified into metabolic pathways (29%), cell cycle control (24%), and cellular components and organization (16%). Molecular functions (middle panel) included: nucleic acid (RNA/DNA) binding proteins (27%), catalytic activity (18%) and protein binding (16%). Cellular components comprised predominated the cytosolic fraction (43%). (**E**) mRNA expression data of ERas Nex binding partners: arginase 1, nucleophosmin, lamin B1, and vimentin on day 0, 1, 4, and 8 of HSC cultivation (n = 3). The error bars indicate S.D.

In spite of sharing a conserved G domain, some members of the Ras family have additional features outside the G domain that may act as functional regulatory modules [35]. The role of the additional 38-amino acid N-terminal extension of ERas is unclear and shows a sequence identity of 42% between human and rat protein (Figure 1A). We have proposed in a mutational study that it may modulate ERas localization through interaction with potential adaptor or scaffold proteins [32]. To find out more about ERas Nex interaction partners, we investigated protein interaction properties of *hs*Nex and *rn*Nex by performing affinity pull-down (n = 3) and MALDI-TOF mass spectrometry using total cell lysates and purified GST-*hs*Nex, GST-*rn*Nex and GST, respectively. Pulldown samples were run on SDS-PAGE and stained with Coomassie brilliant blue. Gel pieces were further processed and analyzed by mass spectrometry as described in Materials and Methods (Figure 1B, white boxes). The bands corresponding to GST-*hs*Nex, GST-*rn*Nex and GST were excluded from the analysis (Figure 1B, black boxes). All proteins interacting with GST-*hs*Nex, GST-*rn*Nex were detected and validated individually with a high degree of confidence based on the peptide sequences using specific databases and programs as described in Materials and Methods. The criteria for considering proteins being significant interactors of ERas Nex included their presence in all three independent pull-down experiments, their absence in the GST pull-down controls, removal of contaminant proteins arising from sample handling (such as keratin and bovine serum albumin). Collectively, we shortlisted a set of 76 ERas Nex potential interacting proteins, 35 of them were associated with *rn*Nex, 21 with *hs*Nex, and 20 proteins were found to bind to both Nex peptides (Figure 1C; Supplementary Table S1).

Identified proteins that interact with ERas Nex were classified into three ontologies: molecular function, biological process, and cellular component (Figure 1D). The vast majority of these proteins are involved in nucleic acid binding, molecular and catalytic activities and protein interactions. They are involved in the control of metabolic processes, cell cycle, and cell communication by localizing in different subcellular compartments, particularly in the cytosol.

Four proteins were selected and analyzed by qRT-PCR (Figure 1E). ARG1 expression was highest in quiescent day 0 HSCs (freshly isolated from rat liver), whereas nucleophosmin and lamin B1 had the highest expression at day 1 and vimentin at day 4 after isolation and initiation of their culture-dependent activation. As we are interested in proteins, that might be important for sustaining the quiescence in HSCs, proteins with a high expression in day 0 cells were of particular interest.

### 3.2. ERas-ARG1 Interaction in Quiescent HSCs

**Figure 2:**
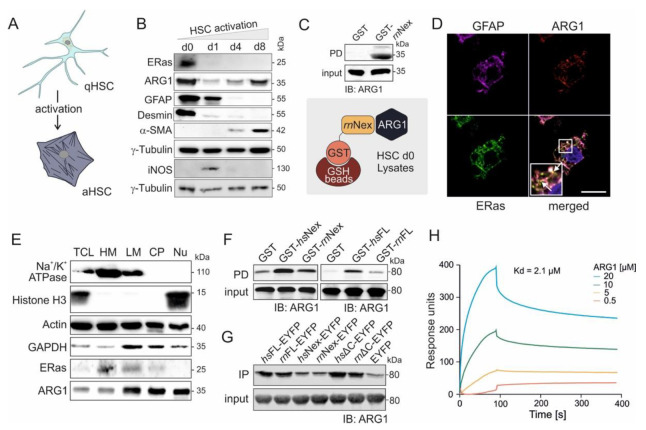
ERas and ARG1 expression, localization, and interaction in hepatic stellate cells. (**A**) Graphic illustration of quiescent (qHSCs) and activated hepatic stellate cells (aHSCs). During culture, the HSCs change in their cell shape and size, and reduce their lipid droplet content. (**B**) Immunoblot analysis of ERas and ARG1 from freshly isolated (d0) and activated HSCs maintained in monoculture for up to 8 days (d1, d4, d8). GFAP was used as a marker for quiescent HSCs (d0), and α-SMA was used as a marker for activated HSCs (d8). γ-tubulin served as a loading control. (**C**) Pull-down assay of ARG1 and GST-*rn*Nex in freshly isolated HSC (d0) lysates. Input and output were immunoblotted and detected with anti-ARG1 antibody. (**D**) Confocal imaging of GFAP, ARG1 and ERas of freshly isolated HSCs after 6 h of culture at day 0 shows colocalization of ERas and ARG1 (white arrows). Scale bar: 10 μm. Six-fold magnification of the merged image. (**E**) Cell fractionation of HSCs d0 in five distinct fractions including: heavy membrane (HM; plasma membrane and rough endoplasmic reticulum), light membrane (LM; polysomes, Golgi apparatus, smooth endoplasmic reticulum), cytoplasm (CP; cytoplasm and lysosomes), nucleus (Nu) and total cell lysate (TCL). Immunoblot analysis was performed for Na^+^/K^+^-ATPase, Histone H3, Actin, GAPDH, ERas and ARG1. (**F**) Pull-down assay of purified ARG1 protein with GST-Nex or GST-FL of human and rat ERas. (**G**) Immunoprecipitation (IP) analysis of ARG1 with various ERas constructs overexpressed in HEK 293 cells (*hs*ERas FL, *rn*ERas FL, *hs*ERas Nex, *rn*ERas Nex, *hs*ERas^ΔC^, *rn*ERas^ΔC^, empty vector). IP was conducted using GFP-coupled nanobeads. Empty EYFP served as a control. Protein complexes retained on the beads were resolved by Laemmli buffer and processed for immunoblotting using a monoclonal antibody against ARG1. (**H**) Sensorgrams obtained from the binding of 0.5–20 µM ARG1 to immobilized GST-*hs*Nex on the surface of a CM5 sensor chip.

In proteomic analysis, we identified ARG1 as a potential binding partner of *hs*Nex and *rn*Nex (Supplementary Table S1), which is also upregulated in quiescent HSCs. Isolated rat HSCs activate during culture on plastic surfaces. Here, they switch from a quiescent state (qHSC) to an activated state (aHSC) (Figure 2A) as they do in response to, for example, chronic liver injury. Day 0 HSCs are considered as quiescent cells, expressing the marker proteins GFAP and desmin, whereas day 8 HSCs are activated and display the activation marker α-SMA. ERas was found to be exclusively expressed at day 0 and also ARG1 exhibited the highest expression at that day 0. The ARG1 expression decreases at day 1 and increases again at day 4 and 8 (Figure 2B). Interestingly, the inducible nitric oxide synthase (iNOS), which uses, like ARG1, L-arginine as substrate, was reciprocally expressed and could only be detected at day 1.

The interaction of ERas and ARG1 was verified by pull-down (Figure 2C) and IP experiments (Supplementary Figure S1), where ARG1 could be pulled down with purified GST-bound ERas *rn*Nex or coprecipitated by fishing with an ERas antibody in freshly isolated HSC d0 cell lysates, respectively. Next, confocal imaging revealed a strong colocalization of ERas and ARG1 in freshly isolated and 6 h cultivated HSCs (Figure 2D, white arrows, additional images in Supplementary Figure S2). The marker GFAP was stained as a quiescent HSC marker. The subcellular localization of ARG1 and ERas was further determined via cell fractionation (Figure 2E). Here, both proteins could be detected in heavy membrane (plasma membrane and rough endoplasmic reticulum), light membrane (polysomes, Golgi apparatus, smooth endoplasmic reticulum) and cytoplasmic fraction (cytoplasm and lysosomes). ARG1 could additionally be found in the nuclear fraction. Furthermore, ARG1 and the Na^+^/K^+^-ATPase colocalized in cLSM pictures as displayed in the Supplementary Figure S3. 

### 3.3. Physical Interaction of ARG1 with ERas

Besides the investigation of protein expression and intracellular localization, we furthermore analyzed the direct interaction of ARG1 and ERas as well as their complex association in vitro. Association of purified ARG1 with different purified ERas variants, including *hs*Nex, *rn*Nex and the full-length ERas orthologs *hs*FL and *rn*FL, was first analyzed in a GST pull-down assay (Figure 2F). Purified GST was used as a control. Figure 2F shows that ARG1 bound much tighter to human than rat ERas proteins. Next, we analyzed the interaction of ARG1 with six human and rat ERas constructs: *hs*FL, *rn*FL, *hs*Nex, *rn*Nex, *hs*ΔC and *rn*ΔC (C-terminal truncated variants), in immunoprecipitation experiments using ERas overexpression in HEK293 cells. Notably, ERas FL and ΔC interaction with ARG1 appeared much stronger as compared to Nex, which indicates that ARG1 might be engaged via additional binding sites other than the N-terminal extension, which contribute to a higher affinity (Figure 2G). Therefore, a pull-down of purified ARG1 with overexpressed N-terminally truncated ERas (∆N; aa 39-233) revealed that ERas ∆N is considerably impaired in binding ARG1 but still exhibited a weak binding as compared to ERas WT and EYFP, which was used as positive and negative controls (Supplementary Figure S4A). This result confirms Nex as a critical binding site of ERas for ARG1 but also confirms that other regions of ERas also contact ARG1. The pull-down of ARG1 with purified GST-ERas G-domain from *rn* and *hs* clearly showed that ERas possess with the G-domain of ERas a second binding site for ARG1 (Supplementary Figure S4B).

Subsequent kinetic of the ERas-ARG1 interaction was investigated using surface plasmon resonance. In this approach, we injected different concentrations of ARG1 (0.5, 5, 10, and 20 µM) to immobilized GST-*hs*Nex (10 µM) and measured their kinetic of interaction (Figure 2H). A dissociation constant (K_d_) of 2.1 µM was determined for GST-*hs*Nex-ARG1 interaction using 1:1 binding Langmuir algorithm model, supporting the previous cell-based analysis of direct protein binding.

### 3.4. Inhibition of ARG1-Polyamine Axis Leads to Accelerated HSC Activation 

**Figure 3:**
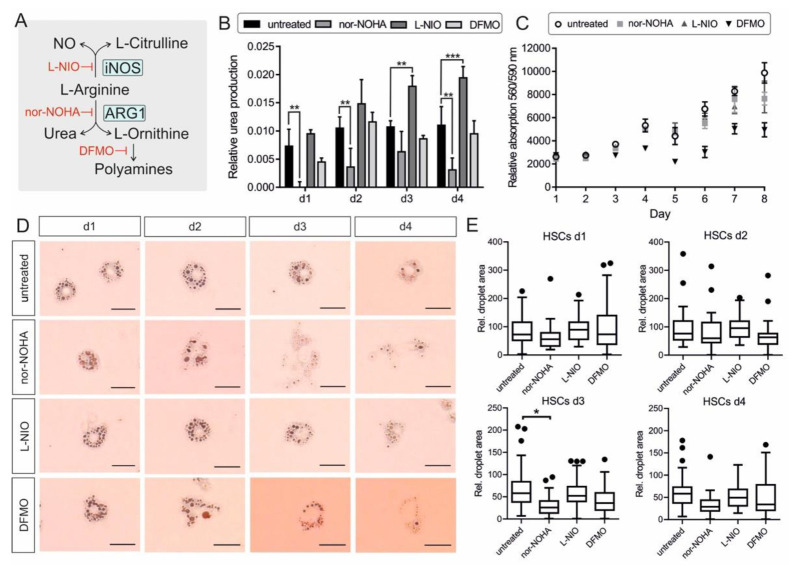
Impact of L-arginine metabolism manipulation on HSC activation. (**A**) Overview of L-arginine metabolism by ARG1 and iNOS and further conversion of L-ornithine to polyamines. Inhibitors of the pathway (iNOS: L-NIO; Arginase: nor-NOHA; ODC1: DFMO) are illustrated in red. (**B**) Urea production of HSCs day 1–4 via colorimetric urea assay in the cell culture supernatants relative to medium control. Significant differences were detected for: untreated—nor-NOHA d1: *p* = 0.00315; d2: *p* = 0.00517; d3: no sig.; d4: *p* = 0.00192/untreated—L-NIO d1: no. sig.; d2: no sig.; d3: *p* = 0.00316; d4: *p* = 0.000934/untreated—DFMO d1-4: no sig. (n = 3) (**C**) Cell viability of HSCs day 0–8 with and without inhibitor treatment via Cell titer blue assay relative to blank medium. The cell viability is correlating proportional with the cell number. (n = 3) (**D**) HSCs at day 1–4 in untreated, nor-NOHA, L-NIO, or DFMO treated conditions and stained with Oil Red O to highlight lipid droplets. The scale bar indicates 0.025 mm. (**E**) Quantitative analysis of ORO-stained droplet number and area (see **D**) of n = 5 cells for each day and condition. Pictures were analyzed by ImageJ and plotted via Prism. The difference between untreated control and nor-NOHA treatment at day 3 was significant (*p* = 0.0308). All statistics were obtained via multiple comparison unpaired *t*-test using Prism 6. The error bars indicate S.D.

In order to investigate the impact of ARG1 activity on HSC quiescence and activation, we chose three inhibitors of the L-arginine metabolism illustrated in Figure 3A. The NO synthase inhibitor N^5^-(1-iminoethyl)-L-ornithine (L-NIO) inhibits the conversion of L-arginine to L-citrulline and NO. N^ω^-hydroxy-L-arginine (nor-NOHA) inhibits the activity of arginase and DL-α-difluoromethylornithine (DFMO) blocks the ornithine decarboxylase I (ODC1), which is the first and rate-limiting enzyme in the polyamine synthesis that converts L-ornithine to putrescine. 

**Figure 4:**
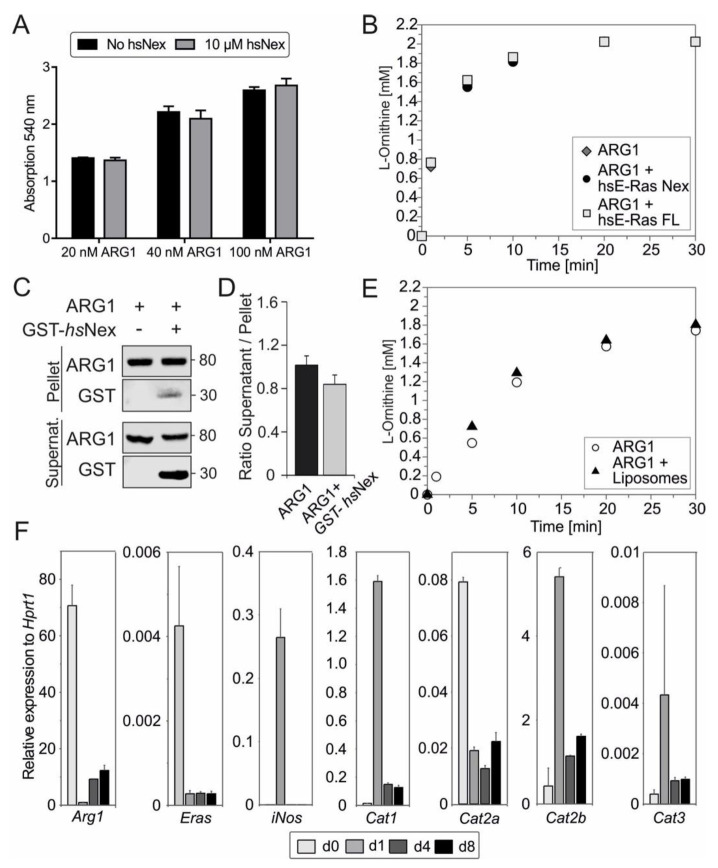
Functional analysis of ERas-ARG1 interaction. (**A**) Arginase activity assay of 20, 40 or 100 nM ARG1, with or without 10 µM *hs*Nex, measured via colorimetric urea assay (n = 3). (**B**) Arginase activity was measured with 2 mM L-arginine, 100 nM ARG1 and 500 nM ERas *hs*Nex or *hs*FL at pH = 9.0 by HPLC. (**C**) Liposome sedimentation assay of 0.2 µM ARG1, immobilized on liposomes together with GST-*hs*Nex and incubated for 30 min at room temperature (RT). After sedimentation with 20,000× *g* for 30 min, immunoblotting was used to show protein binding on liposomes and protein–protein interactions by using monoclonal antibodies against ARG1 and GST. (**D**) Ratio of supernatant to pellet from liposome assay indicating ARG1 binding to liposomes with and without the addition of GST–*hs*Nex. (**E**) Arginase activity measured with 2 mM L-arginine, 100 nM ARG1 without, or in the presence of liposomes at pH = 7.5 in HPLC. (**F**) qRT-PCR analysis of *Arg1, ERas, iNos, Cat1, Cat2a, Cat2b and Cat3* in HSCs at day 0, 1, 4, and 8. Gene expression was normalized to the expression of the housekeeping gene *Hprt1*. All error bars represent S.D.

HSCs were cultured with 0.5 mM L-NIO or nor-NOHA or 1 mM DFMO final concentrations for 4 days. Cell culture supernatant was taken from each condition every day without medium change to determine urea production via a colorimetric urea assay (n = 3). The urea measurement showed a strong increase of urea production and release in the supernatant at day 1 of untreated cells. The urea levels on day 2, 3, and 4 were quite steady. The inhibitory effect of nor-NOHA on ARG1 activity and therefore urea production could be confirmed by almost no detectable urea concentration at day 1 and significantly reduced urea production at day 2 and 4. Interestingly, the NO synthase inhibitor L-NIO caused higher urea levels in the cells compared to all other conditions, most likely due to more substrate availability for arginase. DFMO treatment had no significant effect on the urea level compared to the untreated control (Figure 3B). To confirm that the increased or decreased urea concentrations were not based on a shift in total cell number, a cell viability assay was performed from day 0 to 8 (n = 3). The total cell number did not change up to day 3. At day 4 the cell number increased for untreated, nor-NOHA and L-NIO treated cells, but not DFMO-treated cells. Over the period of 8 days, DFMO-treated cells displayed a considerably lower cell proliferation rate compared to the other conditions, most likely caused by the reduced polyamine concentration in the cells. The addition of nor-NOHA did not change the cell viability and the corresponding cell number (Figure 4C). 

To detect the activation state of the HSCs under the same conditions used earlier, one 24-well was used each day for Oil Red O (ORO) staining, which stains and highlights the lipid droplets within the cytoplasm that get smaller and reduced in number during HSC activation. The ORO staining of the HSCs displayed many big lipid droplets at day 1 for all conditions. The number and diameter of droplets decreased during activation of the untreated cells, still, the cell shape and the characteristic of the lipid droplets showed that the HSCs were not fully activated at day 4. L-NIO treated cells exhibited smaller and more stellate-shaped cell morphology and contained a comparable high amount of lipid droplets at day 4. Nor-NOHA treated cells showed a bulky cell shape from day 3 onwards and a notable loss of lipid droplets. DFMO treated cells exhibited a significant reduction in droplet diameter and a myofibroblast-like cell shape (Figure 4D). The lipid droplet area was used to quantify the state of HSC activation (Figure 4E). At day 1, the cells of all conditions displayed a similar phenotype. At day 3 the difference between untreated cells and arginase inhibited cells was significant (*p* = 0.0308). Due to the limitations of contrast in light microscopy, other factors like cell shape and total cell size could not be considered. An exemplary picture is shown in Supplementary Figure S5, displaying the variable cell morphology of the HSCs with different treatments. The cotreatment of HSCs with nor-NOHA and L-NIO exhibited the phenotype of nor-NOHA treated cells, rather than L-NIO mono-treatment (Supplementary Figure S6).

The results indicated an accelerated activation under nor-NOHA and DFMO treatment, which was absent when L-NIO was added to the HSCs. The data implied that ARG1 activity and the downstream polyamine synthesis impacted on the maintenance of quiescent HSCs, raising the question about the impact of the ERas interaction with ARG1 on its enzyme activity, in this context. 

### 3.5. Human ERas Has No Effect on ARG1 Enzymatic Activity

We next aimed to investigate the impact of ERas on ARG1 enzyme activity. Therefore, we measured the urea production with and without 10 µM ERas *hs*Nex at increasing concentrations of purified ARG1 after one hour (Figure 4A), as well as a full time-dependent cycle of L-arginine conversion into L-ornithine and urea with 100 nM ARG1, 2 mM L-arginine and five times excess ERas *hs*Nex or *hs*FL (0.5 µM) in HPLC at pH 9 (Figure 4B). In both experiments, no change in the velocity of enzymatic ARG1 activity could be detected. Furthermore, we measured fixed ARG1 concentration with increasing concentrations of ERas *hs*FL and *hs*Nex (Supplementary Figure S7) and the Michaelis-Menten constant of the enzymatic reaction of ARG1 (6.5 ± 0.99 mM) and ARG1 + ERas *hs*Nex (7.79 ± 0.33 mM) with concentrations between 0.25 and 10 or 5 mM, respectively (Supplementary Figure S8), and could also not detect any differences. The arginase activity in HSCs was determined by colorimetric urea assay using TCL of HSCs at day 0, 1, 4 and 8 (Supplementary Figure S9). The ARG1 activity in cell lysates was highest in quiescent (d0) HSC lysates, following the protein expression data in Figure 2B.

### 3.6. ARG1 Binding to Liposomes Had No Effect on Its Enzymatic Activity

In a subsequent liposome sedimentation assay, we were able to show ARG1 binding to liposomes, even after applying high forces of 20,000× *g*. In addition, results from this liposome assay indicated the binding of GST-*hs*Nex to ARG1 as seen in immunoblotting with GST-antibody (Figure 4C). GST was used as a negative control (data not shown). The ratio of supernatant to the pellet of ARG1 binding with and without the presence of ERas *hs*Nex does not display significant changes (Figure 4D). In order to investigate a possible function of membrane binding towards the enzymatic activity of ARG1, a full enzymatic reaction of ARG1 and 2 mM L-arginine was performed in the presence of liposomes at pH 7.5 (Figure 4E). This experiment did not show any major differences as well. Taken together, the interaction of ERas and ARG1, but also the association of ARG1 with the membrane does not contribute to an enhanced enzymatic activity of ARG1. 

### 3.7. Correlation of ERas, Arg1, and Cat2a mRNA Expression in qHSCs

In the next approach, we determined the expression of different L-arginine transporters in quiescent and activating HSCs (Figure 4F). Even though ERas interaction with ARG1 does not affect the enzymatic activity of the latter, the interaction between the two proteins might be important for the subcellular localization of ARG1 or the formation of a functional microdomain with, for example, L-arginine transporter at the plasma membrane. Therefore, mRNA levels were determined by qRT-PCR, showing the same pattern for mRNA and protein expression (Figure 2B and Figure 4F) for *Arg1, Eras* and *iNos*. Interestingly, the cationic amino acid transporter (CATs) with the paralogs CAT1, CAT2A, CAT2B and CAT3 were regulated strongly during HSC activation. *Cat1* mRNA expression was highest at day 1, correlating with iNOS expression. *Cat2a* mRNA expression showed the highest expression in quiescent HSCs at day 0 and reduced expression in more activated states of HSCs, equivalent to *Arg1* and *ERas* regulation. The mRNA level of the CAT isoform 2B was upregulated at day 1 and was the highest among all paralogs. In contrast, *Cat3* mRNA expression was the lowest in comparison to the other paralogs and was slightly upregulated at day 1. The interaction or colocalization of ARG1, ERas and CAT2B could not be investigated yet due to the insufficient quality of the CAT2 antibody, but will provide an interesting research approach for the future.

## 4. Discussion

The aim of this study was to identify new interaction partners of ERas that are involved in the maintenance of HSC quiescence. Hepatic stellate cells are not only important to sense changes within their stem cell niche, but also to contribute to inflammatory events and communicate with neighboring cells. Their role in the pathological changes during liver fibrosis is well known and connected to the continuous activation of quiescent HSCs resulting in extracellular matrix-producing myofibroblast-like cells. Therefore, identification of signaling pathways and specific proteins that are essential for HSCs quiescence or activation is necessary to understand the molecular basis of liver fibrosis and development of therapeutic approaches for patients with chronic liver diseases. 

In this study, we characterized the protein binding properties of the unique N-terminal extension of ERas, which is 42% identical between rat and human proteins. The MS analysis identified 20 shared proteins, pulled down with the rat and human N-terminal region (Nex), as well as 35 proteins for *rn*Nex alone and 21 proteins for *hs*Nex. The molecular functions of ERas interaction partners are diverse and reach from nucleic acid binding, like the protein nucleophosmin, and structural molecule activity, like vimentin, to catalytic activities or translation regulation. Therefore, biological processes are widespread with a tendency of 29% of metabolic processes. This category includes ARG1 with its L-arginine-hydrolyzing enzymatic activity. The cellular localization of ERas binding partners are predominantly cytosolic, which coincides with the heavy and light membrane localization of ERas due to its post-translational modifications by farnesylation and palmitoylation [8,32]. We checked the mRNA expression of four representative proteins, that differ strongly in localization and function. ARG1 was expressed strongly at day 0 cells, which are considered as quiescent HSCs. The expression of ARG1 got downregulated during activation, which gave us a first hint of the involvement of ARG1 in the maintenance of HSC quiescence. 

In cell biological experiments, we could confirm the coexpression, colocalization, and direct interaction of ERas and ARG1 in quiescent HSCs and also in cell-free experiments using purified proteins. Furthermore, we could confirm an active role of ARG1 when HSCs become myofibroblast-like cells, as the inhibition of arginase by nor-NOHA accelerated the activation of primary rat HSCs. Similar results were obtained after using the ODC1 inhibitor DFMO during HSC cultivation. These data suggest that not only the involvement of ARG1, but also the downstream production of polyamines are of major importance to maintain quiescence in HSCs. Polyamines can get synthesized downstream of L-ornithine by the ODC1 and further reversibly processed from putrescine to spermidine and spermine. The roles of polyamines in cells are broad and vary from cell proliferation to gene expression, ion channel regulation, and protection from oxidative damage to autophagy and regulatory aspects in immune cells [36,37,38,39]. Other studies using carbon tetrachloride (CCl_4_) or ethanol for inducing liver injury demonstrated a protective effect of polyamines as well as a positive effect on hepatocyte proliferation [40,41]. Interestingly, it was also reported that polyamines support the self-renewal of embryonic stem cells (ESCs) [42,43]. ERas is highly expressed in ESCs, still ARG1 could neither be detected in the mRNA profile of embryonic stem cells, nor in mesenchymal stem cells (Supplementary Figure S10). However, polyamines can be transported from and to other cells via transporter systems and do not necessarily need to be produced and consumed in the same cell [44]. Even though the effect of polyamines on HSCs has not been described, it is tempting to speculate that the communication of HSCs and hepatocytes might include the production and exchange of polyamines to maintain liver homeostasis and regeneration after tissue injury. This hypothesis needs to be verified experimentally in future studies.

The interaction of ERas and ARG1 did not show any effects on ARG1 enzymatic activity in our experimental set-up. Therefore, the interaction of ERas and ARG1 may have other functional significance that can only be speculated about at this point. On the one hand, ARG1 and iNOS, which are reciprocally expressed during HSC activation, might each directly control the other’s expression. The elevated expression of iNOS, one day after isolation and cultivation outside of their stem cell niche, is likely to be a stress response, driven by the transcription factor NFκB that regulates iNOS mRNA expression [45]. NFκB was furthermore described to play an essential role in both profibrogenic and antifibrogenic signaling pathways during liver diseases, and was also investigated in HSCs [46,47]. The expression level of iNOS is not only controlled by NFκB, but also by the translation initiation factor 2α (eIF2α), which is in turn regulated by L-arginine levels and therefore likely to be connected to ARG1 activity, also shown in astrocytes which have multiple similarities with HSCs [48,49,50]. On the other hand, the interaction of ERas and ARG1 could recruit ARG1 to a specific subcellular localization on lipid membranes to either increase ARG1 activity as it was shown in red blood cells, where the activity of arginase was approximately 100 times higher in membrane fractions compared to cytoplasmic fractions [51], or to source exogenous but not endogenous L-Arg, according to observations in other cells types, thereby solving “the arginine paradox” [52]. ERas-ARG1 interaction might recruit ARG1 to the plasma membrane close to a cationic amino acid transporters (CATs) whose paralogs considerably differ in their affinity for L-arginine (CAT1: 0.10–0.16 mM; CAT2A 2–5 mM; CAT2B and CAT3: 0.25–0.70 mM) [53]. One hint might be the coexpression of ARG1 and CAT2A in quiescent HSCs that have a comparable low affinity for L-arginine and would match consumption and transportation affinity of L-arginine. For comparison, iNOS is coexpressed with the inducible CAT2B paralog in HSCs at day 1, which has also been reported for astrocytes [49,54]. In order to analyze the formation of an ARG1-ERas-CAT2A colocalization on the plasma membrane, future extensive studies and validated antibodies, which we do not currently have, are needed. 

This study could successfully add arginase 1 to the list of interaction partners of ERas and to the proteins that are needed for the maintenance of quiescent HSCs. The alteration of the L-arginine metabolism *via* arginase and ODC1 inhibitors shows a strong picture towards an accelerated activation, whereas the iNOS inhibitor slows down the development of HSCs into myofibroblast-like cells. As HSCs are located in a tightly controlled stem cell niche in the space of Disse [10], we always have to consider the effect of amino acid/polyamine depletion and delivery, as well as the communication of HSCs as liver pericytes with surrounding cells and the vascular system.

## Data Availability

This study includes no data deposited in external repositories.

## Supplementary Materials

The following are available online at https://www.mdpi.com/article/10.3390/cells11030508/s1, Figure S1: Immunoprecipitation analysis of ARG1 and *rn*ERas, Figure S2: Confocal images of ERas, ARG1 and GFAP in quiescent HSCs., Figure S3: Confocal images of ERas—ARG1 and ARG1—Na^+^/K^+^-ATPase in quiescent HSCs., Figure S4: Pull-down experiments of ERas *hs*FL, *hs*∆N and *rn*/*hs* G-domain with purified ARG1., Figure S5: Phase contrast microscopy of HSCs day 4., Figure S6: Oil Red O staining of HSCs day 1 to 4 with combination treatment of L-NIO and nor-NOHA., Figure S7. Urea assay with ARG1 and human ERas FL or Nex., Figure S8. ARG1 kinetic with and without ERas hsNex., Figure S9: Colorimetric arginase activity assay in HSC lysates., Figure S10: qRT-PCR analysis of ARG1 and OCT4 in HSCs, ESCs and MSCs., Table S1: Interaction partners of rat and human ERas Nex., Table S2. Primary and secondary antibodies. Table S3. qRT-PCR Primers.
