# Supplementary material for: Physical Interaction between Embryonic Stem Cell-Expressed Ras (ERas) and Arginase-1 in Quiescent Hepatic Stellate Cells
Source: Cells. 2022 Feb 1;11(3):508. doi: 10.3390/cells11030508 (PMC8834437; doi:10.3390/cells11030508)
Supplement: Supplementary file 1 [file cells-11-00508-s001.zip › cells-1519927-supplementary.pdf]

Supplementary Information

Physical interaction between embryonic stem cell-expressed Ras (ERas) and Arginase-1 in quiescent hepatic stellate cells

S. Pudewell, J. Lissy, H. Nakhaeizadeh, M.S. Taha, M. Akbarzadeh, S. Rezaei Adariani, S. Nakhaei-Rad, J. Li, C. Kordes, D. Häussinger, R.P. Piekorz, M.M. Cortese-Krott, M.R. Ahmadian

Institute of Biochemistry and Molecular Biology II, Medical Faculty and University Hospital Düsseldorf, Heinrich Heine University Düsseldorf, Düsseldorf, Germany

Table S1. Interaction partners of rat and human ERas Nex.

Protein name (gene name)	Function	MW (kDa)	p-values *Hs*/*Rn*	MS/MS count	Unique peptide	Seq. cov. (%)	Acc. ID
40S ribosomal protein S2 (RPS2)	Translation-	31.3	0.231/0.303	8	3	14.3	P15880
60S ribosomal protein P0 (RPLP0)	Translation	34.3	0.921/0.545	24	7	36.6	P05388
78 kDa ER chaperone (HSPA5)	Chaperone	72.3	0.175/0.651	191	24	45.6	P11021
Alpha cardiac muscle 1 (ACTC1)	Actin filament	42.0	0.943/0.313	8	5	53.6	P68032
Actin, cytoplasmic 2 (ACTG1)	Actin filament	41.8	0.783/0.407	253	2	74.4	P63261
ADP/ATP translocase 3 (SLC25A6)	Apoptosis	32.9	0.097/0.010	4	2	19.5	P12236
Alpha-actinin-4 (ACTN4)	Actin filament	104.9	0.577/0.732	57	15	35.8	O43707
Annexin A2 (ANXA2)	Vesicle budding	38.6	0.537/0.112	47	11	35.4	P07355
Arginase-1 (ARG1)	Metabolism	34.8	0.810/0.879	22	6	22	P05089
ATP-dep. RNA helicase A (DHX9)	Transcription	141.0	0.670/0.565	49	21	23.9	Q08211
ATP-dep. RNA helicase (DDX3X)	Immune response	73.2	0.947/0.922	25	9	23.6	O00571
Barrier-to-autointegration factor (BANF1)	Nuclear transport	10.1	0.649/0.305	9	2	40.4	O75531
Caldesmon (CALD1)	Actin binding	93.2	0.955/0.261	8	5	13	Q05682
Calmodulin-like 5 (CALML5)	Ca^{2+} binding	15.9	0.235/0.623	25	5	38.4	Q9NZT1
Caprin-1 (CAPRIN1)	Translation	78.4	0.114/0.370	11	5	10.7	Q14444
Carbonyl red. [NADPH] 1 (CBR1)	Differentiation	30.4	0.415/0.753	500	19	74	P16152
Cathepsin D (CTSD)	Proteolysis	44.6	0.151/0.020	4	3	11.7	P07339
Caveolae-associated protein 1 (CAVIN-1)	Transcription	43.5	0.507/0.038	5	3	16.7	Q6NZI2
Core histone macro-H2A.1 (H2AFY)	Transcription	39.6	0.129/0.141	10	4	20.9	O75367
Corneodesmosin (CDSN)	Cell adhesion	51.5	0.930/0.845	9	3	8.9	Q15517
C-terminal-binding protein 2 (CTBP2)	Proliferation	48.9	0.335/0.042	7	4	12.4	P56545
Cystatin-A (CSTA)	Cell adhesion	11.0	0.500/0.688	63	6	67.3	P01040
Dermcidin (DCD)	Defense response	11.3	0.371/0.375	87	3	22.7	P81605
Desmocollin-1 (DSC1)	Ca^{2+} binding	100.0	0.405/0.725	44	5	8.3	Q08554
Desmoglein-1 (DSG1)	Cell adhesion	113.7	0.167/0.136	139	17	25.8	Q02413
Desmoplakin (DSP)	Cell adhesion	331.8	0.258/0.293	147	34	14.7	P15924
Drebrin (DBN1)	Actin binding	71.4	0.693/0.976	22	10	31.3	Q16643
Electron transfer flavoprotein subunit alpha, mitochondrial (ETFA)	Oxidoreductase activity	35.1	0.477/0.220	35	1	6.7	P13804
Elongation factor 1-beta (EEF1B2)	Translation	24.8	0.590/0.119	11	3	20.4	P24534
Enhance of mRNA-decapping protein 4 (EDC4)	mRNA degradation	151.7	0.000/0.923	47	14	17.2	Q6P2E9
Epiplakin (EPPK1)	Interm. filament	555.7	0.996/0.877	81	30	32.7	P58107
Fibronectin (FN1)	Matrix protein	262.6	0.644/0.514	19	8	7.6	P02751
Filamin-A (FLNA)	Actin binding	262.6	0.737/0.210	167	46	30.2	P21333
General transcription factor II-I (GTF2I)	Transcription	112.4	0.274/0.491	27	7	14	P78347
Glyceraldehyde-3-phosphate dehydrogenase (GAPDH)	Metabolism	36.1	0.644/0.315	14	4	20.5	P04406
Heat shock 70 kDa protein 1B (HSPA1B)	Chaperone	70.1	0.757/0.685	169	8	49.3	P0DMV9
Heat shock 71 kDa protein (HSPA8)	Chaperone	70.9	0.911/0.943	352	25	62.7	P11142
Heterogen. nuclear RNP A1 (HNRNPA1)	mRNA transport	38.7	0.245/0.053	15	6	33.3	P09651
Histone H1.2 (HIST1H1C)	Transcription	21.4	0.473/0.945	29	2	27.2	P16403
Importin subunit beta-1 (KPNB1)	Mitosis	97.2	0.626/0.413	22	4	6.3	Q14974
Interleukin enhancer-binding factor 2 (ILF2)	Transcription	43.1	0.893/0.952	11	4	17.4	Q12905

Junction plakoglobin (JUP)	Cell adhesion	81.7	0.382/0.090	122	16	29.9	P14923
Lamin-B1 (LMNB1)	Nuclear lamina	66.4	0.276/0.164	21	10	30.5	P20700
LIM domain/actin-binding protein 1 (LIMA1)	Actin binding	85.2	0.435/0.379	25	8	27.6	Q9UHB6
Matrin-3 (MATR3)	Immune response	94.6	0.344/0.945	35	10	19	P43243
Methylosome subunit pICln (CLNS1A)	RNA splicing	26.2	0.191/0.608	59	3	27.8	P54105
Myb-binding protein 1A (MYBBP1A)	Transcription	148.9	0.337/0.271	22	11	11.4	Q9BQG0
Myosin-9 (MYH9)	Motor activity	226.5	0.549/0.862	44	14	12.2	P35579
Neuroblast differentiation-associated protein AHNAK (AHNAK)	RNA splicing	692.1	0.475/0.272	43	29	13.7	Q09666
Nuclear mitotic app. protein 1 (NUMA1)	Meiotic cell cycle	238.3	0.349/0.099	51	24	17.3	Q14980
Nucleolar protein 56 (NOP56)	rRNA processing	66.1	0.275/0.091	15	7	19.4	O00567
Nucleolar RNA helicase 2 (DDX21)	Transcription	87.3	0.615/0.609	44	14	27.5	Q9NR30
Nucleolin (NCL)	Translation	76.6	0.446/0.504	13	7	15.2	P19338
Nucleophosmin (NPM1)	Transcription	32.6	0.210/0.602	73	10	44.9	P06748
Peroxiredoxin-1 (PRDX1)	Proliferation	22.1	0.928/0.406	28	4	33.2	Q06830
Plectin (PLEC)	Actin binding	531.8	0.584/0.373	508	2	43.8	Q15149
Polypyrimidine tract-bind. protein 1 (PTBP1)	mRNA processing	57.2	0.463/0.171	15	5	17.7	P26599
Prelamin-A/C (LMNA)	Nuclear lamina	74.1	0.485/0.241	86	25	47.3	P02545
Probable 28S rRNA (cytosine(4447)-C(5))-methyltransferase (NOP2)	Ribosomal large subunit assembly	89.3	0.047/0.222	8	6	12.1	P46087
ATP-dependent RNA helicase (DDX5)	Transcription	69.1	0.149/0.136	23	5	21.2	P17844
Prolactin-inducible protein (PIP)	Host defense	16.6	0.877/0.568	26	5	49.3	P12273
Protein S100-A7 (S100A7)	Immune response	11.5	0.536/0.469	12	2	22.8	P31151
Protein-glutamine γ-glutamyltransferase E (TGM3)	Ca^{2+} binding	76.6	0.301/0.196	18	4	10.1	Q08188
Elongation factor 1-α-like 3 (EEF1A1P5)	Translation	50.2	0.316/0.903	23	4	16.2	Q5VTE0
RuvB-like 2 (RUVBL2)	DNA repair	51.2	0.367/0.165	3	3	8.6	Q9Y230
SAFB-like transcription modulator (SLTM)	Apoptosis	117.2	0.183/0.286	8	3	3.1	Q9NWH9
Scaffold attachment factor B1 (SAFB)	Transcription	102.6	0.063/0.444	11	3	10	Q15424
Small proline-rich protein 2A (SPRR2A)	Keratinocyte diff.	8.0	0.126/0.623	3	2	43.1	P35326
Splicing factor 3A subunit 1 (SF3A1)	mRNA processing	88.9	0.549/0.884	4	4	10.1	Q15459
Stress-70 protein, mitochondrial (HSPA9)	Differentiation	73.7	0.389/0.907	108	19	39.3	P38646
SWI/SNF-related matrix-ass. actin-dep. regulator of chromatin A5 (SMARCA5)	Transcription	121.9	0.126/0.001	5	3	7.3	O60264
Tubulin alpha-1C chain (TUBA1C)	Microtubules	49.9	0.974/0.136	23	5	16.9	Q9BQE3
Tyrosine-protein kinase (BAZ1B)	Transcription	170.9	0.737/0.558	7	6	5.9	Q9UIG0
Ubiquitin-60S ribo. protein L40 (UBA52)	Translation	14.7	0.336/0.810	62	5	38.3	P62987
Vimentin (VIM)	Cytoskeleton	53.7	0.935/0.379	379	45	78.1	P08670
Zinc-alpha-2-glycoprotein (AZGP1)	Cell adhesion	34.3	0.589/0.633	17	4	18.5	P25311

Table S2. Primary and secondary antibodies.

Protein	Host	Company	Product No°
α-SMA	mouse	Dako	M0851
actin	mouse	Merck	MAB1501
ARG1	goat	Santa Cruz	sc-18351
Desmin			
ERas clone 6.5.2		Own antibody	
FLAG	rabbit	Sigma Aldrich	F7425
GAPDH	rabbit	Cell Signaling	2118
GFAP	rabbit	Dako	Z0334
GST		Own antibody	
γ-tubulin	mouse	Merck	T5326
iNOS	mouse	BD Bioscience	610328
Na+/K+ ATPase	mouse	Merck	A276
**Secondary Immunoblotting antibodies**			
IRDye® 800CW anti-Rabbit IgG	donkey	Licor	926-32213
IRDye® 680 RD anti-Mouse IgG	donkey	Licor	926-68072
IRDye® 800CW anti-Goat IgG	donkey	Licor	926-32214
**Secondary cLSM antibodies**			
Alexa Fluor 488- conjugated anti-mouse IgG	goat	Thermo Fisher Scientific	A32723
Alexa Fluor 546-conjugated anti-goat IgG	donkey	Thermo Fisher Scientific	A11056
Alexa Fluor 633-conjugated anti-rabbit IgG	goat	Thermo Fisher Scientific	A21070

Table S3. qRT-PCR Primers.

Target	Forward Primer	Reverse Primer
rn Arg1	FW 5'-TTGGGTGGATGCTCACACTG-3'	RV 5'-GTACACGATGTCCTTGGCAGA-3'
rn CAT1	FW 5'-GCCATCGTCATCTCCTTCCTG-3'	RV 5'-CCCTCCCTCACCGTATTTCAC-3'
rn CAT2A	FW 5'-CCTTACCCCGCATTCTGTTTG-3'	RV 5'- AAATGACCCCTGCAGTCATCG-3'
rn CAT2B	FW 5'-GCTCCCTCTGCGCCTTATCA-3'	RV 5'-CATCACAGCTGCCACTGCAC-3'
rn CAT3	FW 5'- CACAGAACCGCACTCCCCTT-3'	RV 5'-GCATCCAGACCCCAAATCGG-3'
rn Hprt1	FW 5'-AAGTGTTGGATACAGGCCAGA-3'	RV 5'-GGCTTTGTACTTGGCTTTTCC-3'
rn iNOS	FW 5´-TGGTGAGGGGACTGGACTTTT-3´	RV 5´-TTCTCCGTGGGGCTTGTAGT-3´
rn LMNB1	FW 5´-CCGGGCTCAAGGCTCTCTA-3'	RV 5´-GCGCGGCCTCATACTCTC-3'
rn NPM1	FW 5´-AACTCTTAGGCATGTCTGGAAAG-3'	RV 5´-GCTGGGGTATCTCGTACAGATTT-3'
rn VIM	FW 5´-CGGCTGCGAGAAAAATTGC-3'	RV 5´-CCACTTTACGTTCAAGGTCAAG-3'

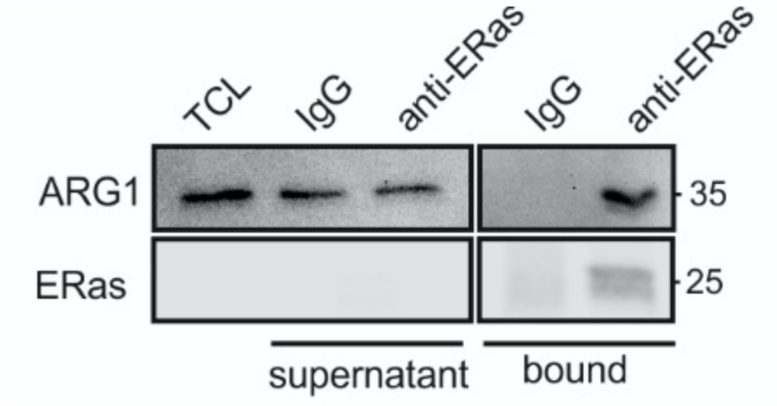

Figure S1. Immunoprecipitation analysis of ARG1 and *rn*ERas. IP in HSC day 0 lysates with monoclonal antibody against ERas, following immobilized on Protein G beads. Proteins retained on the beads were resolved by Laemmli buffer and processed for Western blot using a monoclonal antibody against ARG1.

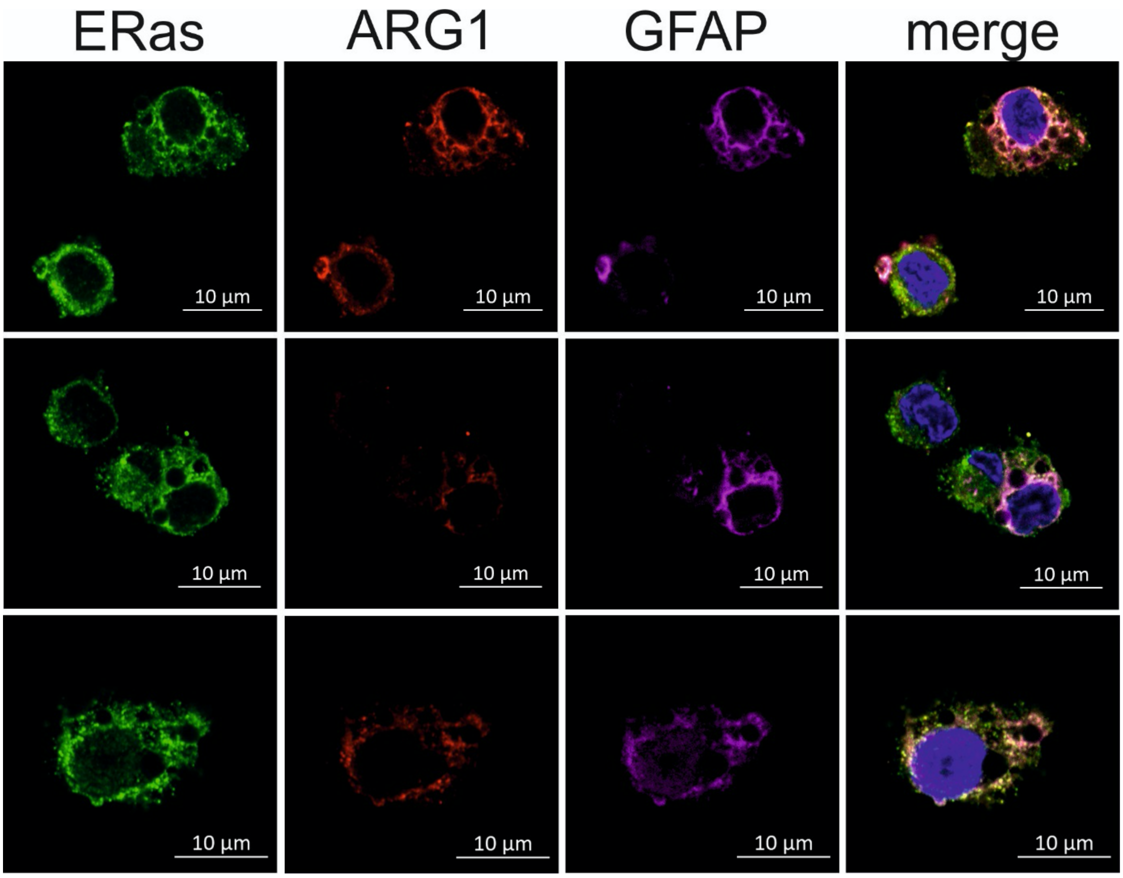

Figure S2. Confocal images of ERas, ARG1 and GFAP in quiescent HSCs. ERas was stained in green, ARG1 in red and GFAP in pink. The conditions are same as for the pictures obtained in Figure 2D and described in material and methods. The scale bar indicates 10 µm. Co-localization is displayed in the merged picture in the white areas (overlapping signals).

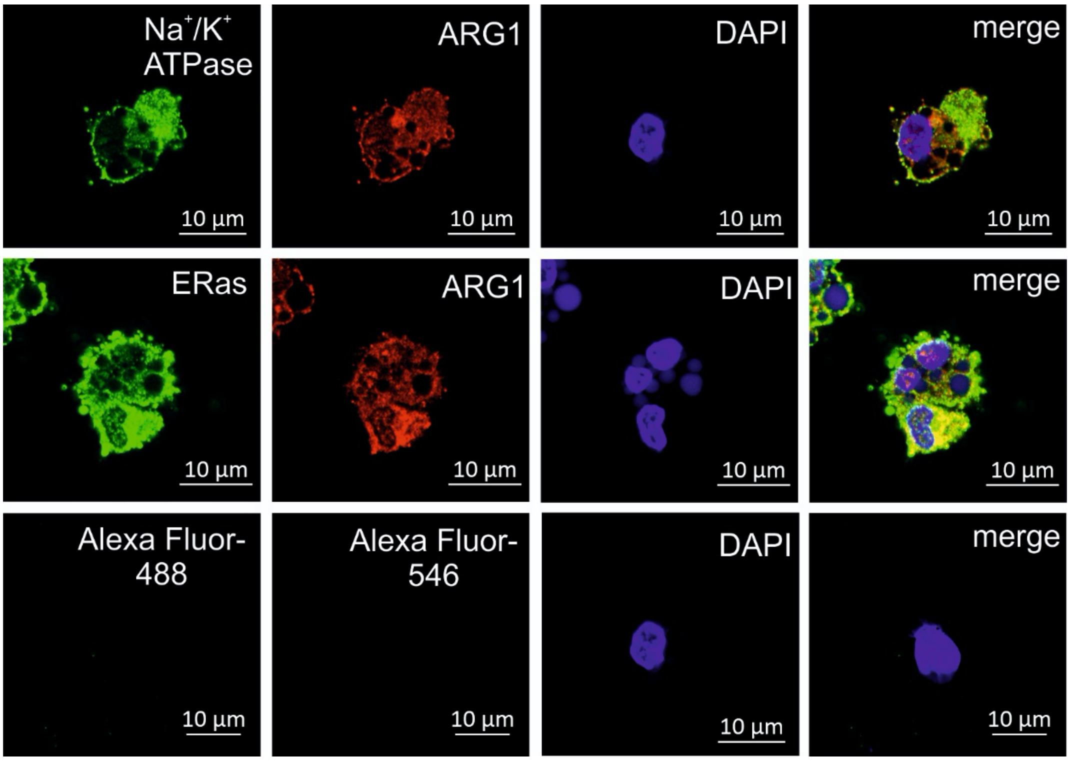

Figure S3. Confocal images of ERas - ARG1 and ARG1 - Na^+/K^+-ATPase in quiescent HSCs. Co-staining of ARG1 and Na^+/K^+-ATPase is displayed in the first row, ERas and ARG1 in the second row and the negative control of the secondary antibodies alone in the third row. Merged pictures are located in the very right column. Freshly isolated HSCs were seeded on cover slips and cultivated for 6 hours. The staining procedure was performed following the conditions in material and methods. Antibodies used were: ERas 6.5.2 (own antibody), mouse, dilution: 1:40; ARG1 #HPA003595, Sigma, rabbit, dilution 1:100 and Na^+/K^+-ATPase # ab283318, abcam, mouse, dilution: 1:100. The secondary antibodies are mentioned in the Supplementary table S2.

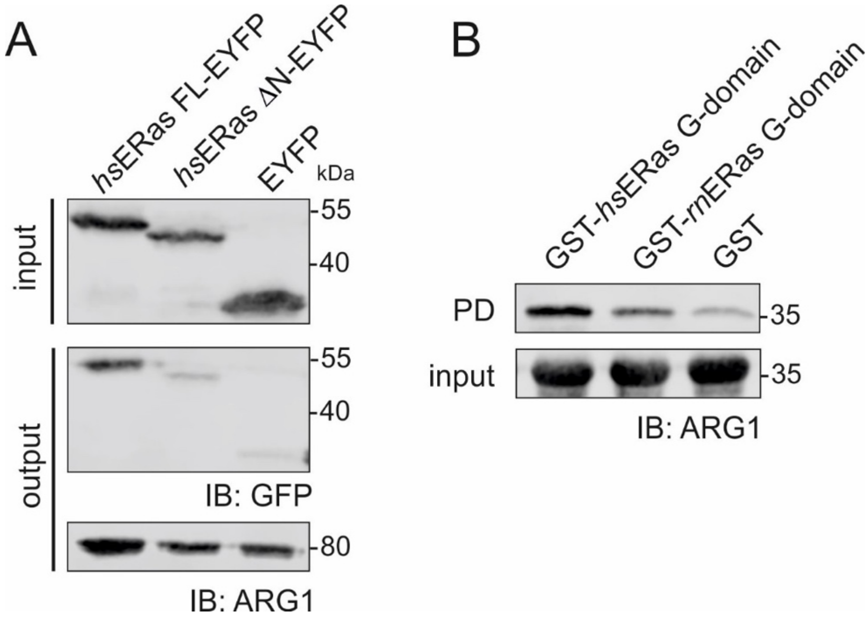

Figure S4. Pull down experiments of ERas *hs*FL, *hs*ΔN and *rn/hs* G domain with purified ARG1. (A) Pull-down analysis of MBP-ARG1-His with overexpressed hsERas FL, ΔN (aa 39-233) and empty vector pEYFP in HEK 293T cells. His Mag Sepharose® Ni beads from Cytiva were incubated with 10 µg purified MBP-ARG1-His for 1h at 4°C. After washing 3 times with binding buffer (20 mM Tris, 500 mM NaCl, 20 mM Imidazol), the beads were incubated with HEK lysates of EYFP-tagged ERas FL, ERas ΔN or empty EYFP overexpression (one 10 cm dish each). Beads were washed and remaining proteins were resuspended in 80 µl 1x Laemmli. Samples were run on a 10 % SDS gel and immunoblotted using an anti-ARG1 (#sc-166920, Santa Cruz, mouse) and anti-GFP (#PA1-980, invitrogen, rabbit) antibody. ERas ΔN binds much weaker to ARG1 than ERas-FL. EYFP was used as a negative control. (B) Pull-down analysis of GST-ERas G-domain (*rn* or *hs*) and ARG1-His (*hs*). GST and GST-ERas proteins, overexpressed in *E. coli*, were isolated using GSH beads. The beads were incubated with purified ARG1-His for 30 min on ice. The beads were centrifuged, washed and ultimately mixed with Laemmli buffer. Samples were run on a 12.5 % SDS gel and immunoblotted using an anti-ARG1 (#sc-18351, Santa Cruz, goat) antibody. A faint band of ARG1 was detected for GST alone which was used as a negative control.

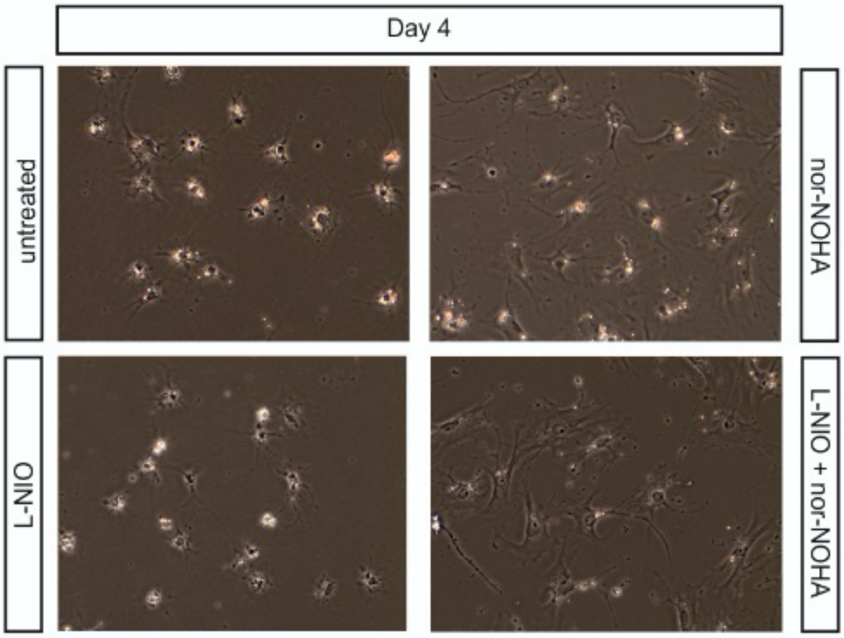

Figure S5. Phase contrast microscopy of HSCs day 4. Untreated, L-NIO, nor-NOHA and combination L-NIO and nor-NOHA treatment at day 4 after isolation (0.5 mM final concentration each). Phase contrast shows the cell morphology stronger. Elucidated spots indicate lipid droplets. Pictures were taken with 40x magnification, without cropping the pictures.

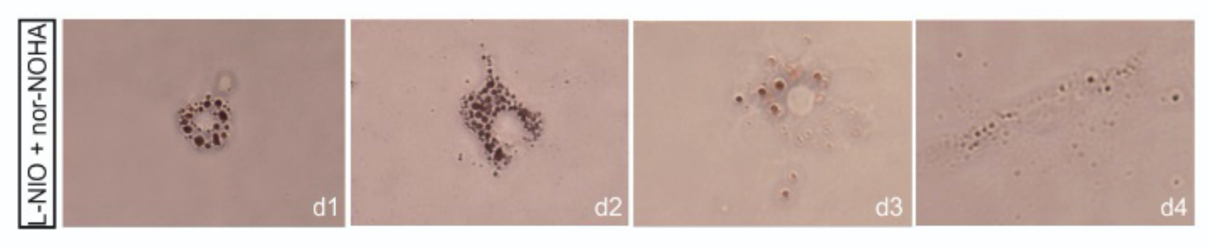

Figure S6. Oil Red O staining of HSCs day 1 to 4 with combination treatment of L-NIO and nor-NOHA. Dark red spots indicate lipid droplets. Cell activation can be analyzed by the number, size and structure of lipid droplets. Both inhibitors were used with 0.5 mM final concentration. Pictures were taken with 40x magnification and cropped afterwards.

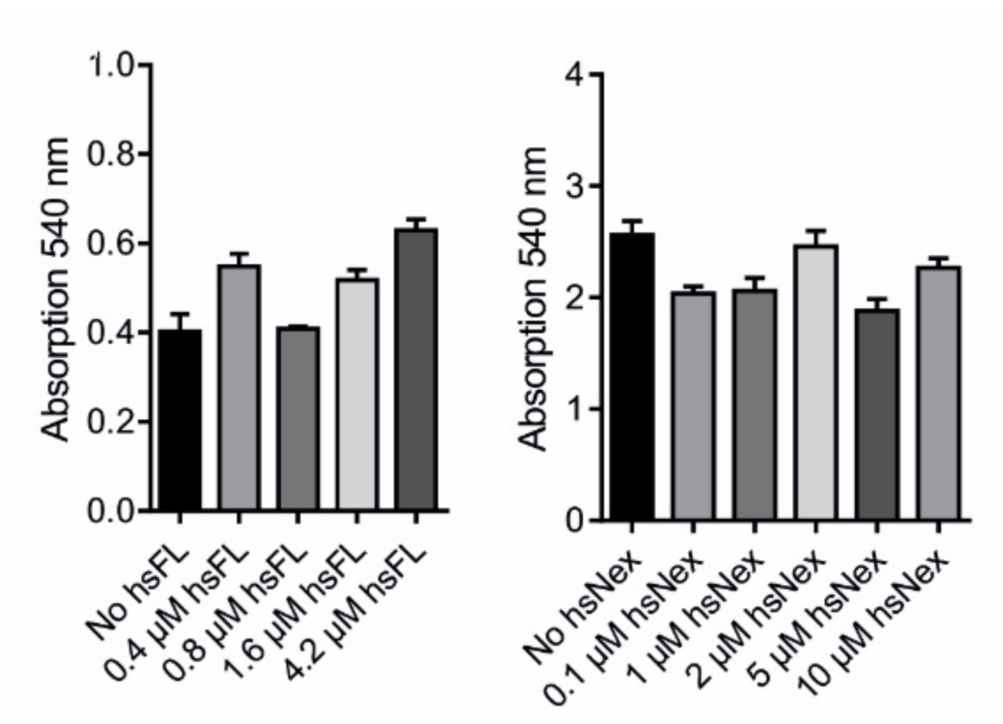

Figure S7. Urea assay with ARG1 and human ERas FL or Nex. Colorimetric urea assay of 20 nM ARG1 and different concentrations of human ERas FL (0.4, 0.8, 1.6 and 4.2 µM) (left), or ERas Nex (0.1, 1, 2, 5, 10 µM) (right). The samples were incubated for 1 hour at 37 °C. The readout was performed at 540 nm in a TECAN plate reader. Data displayed with ±SD.

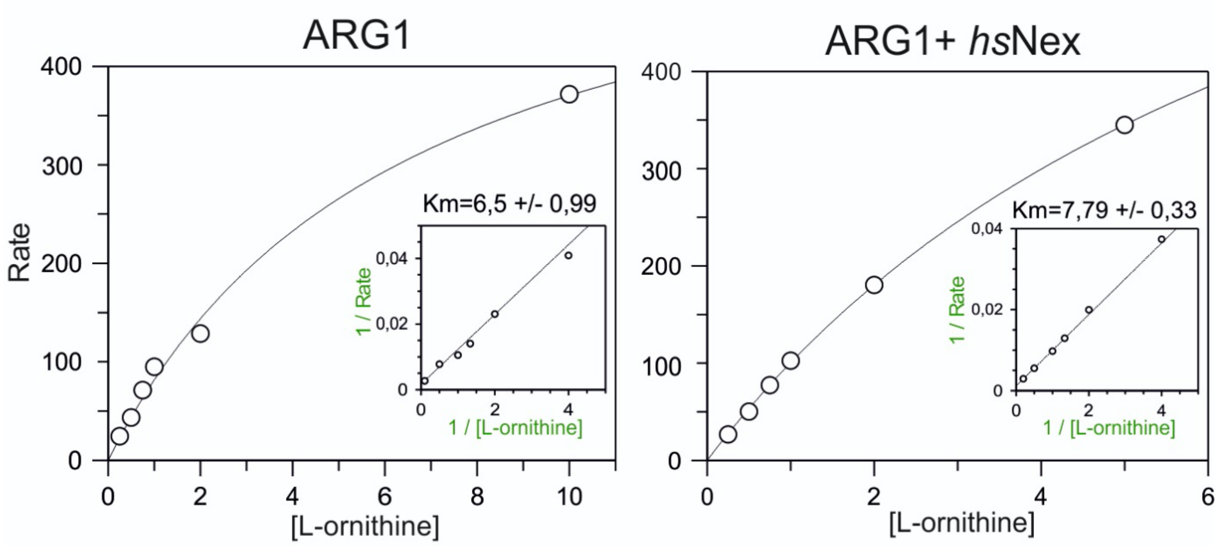

Figure S8. ARG1 kinetic with and without ERas hsNex. LC-MS analysis of 100 nM ARG1 with different concentrations L-arginine (0.25, 0.5, 0.75, 1, 2, 10 mM for ARG1 alone or 0.25, 0.5, 0.75, 1, 2, 5 mM for ARG1 with hsNex) at 15 sec, 1 min, 3 min and 5 min time points. The slope of the reaction was plotted in a Michaelis-Menten graph. The calculated Km was 6.5 ± 0.99 for ARG1 and 7.79 ± 0.33 for ARG1 with ERas hsNex.

Method: ***Chemicals and reagents*** - L-arginine (Arg), were purchased from Sigma-Aldrich (Gillingham, Dorset, UK). Labelled internal standard L-Arginine-$^{13}C_6$ hydrochloride was obtained from Sigma-Aldrich. All other chromatography-grade chemicals such as acetonitrile and formic acid were purchased from Sigma-Aldrich. The deionized water (Milli-Q) was used to prepare the solutions. ***Sample preparation*** - Calibrator of L-arginine was prepared for 1 mM in stock and stored at - 20 °C before use. For the calibration, stock solution was mixed and diluted into 0.2 µM to 200 µM for the analysis. Samples with L-Arg and ARG1 substrates were prepared as described in 2.7 and diluted before transferred into glass vial with an inner tube for further detection by LC-MS. All the calibrators and samples were spiked with 10 µM of ^{13}C-labelled internal standard before analysis, where the calibrators were measured in triplicate and samples in duplicate accordingly. ***Instrumentation*** - The analyses of the amino acid standards and samples were performed on an Agilent 1290 Infinity UHPLC system coupled to a 6550 QTOF-MS (Agilent Technologies, Waldbronn, Germany), equipped with a Dual Agilent Jet Stream Electrospray Ionization (Dual AJS - ESI) source. The positive ionization mode was applied for the detection with gas temperature at 200 °C; drying gas flow of 12 L/min; nebulizer for 35 psig; sheath gas temperature at 330 °C and flow of 11 L/min. The scan range was from 100 to 1000 m/z. ***HPLC conditions*** – 5 µl of standards or samples were injected to the LC system separated on a Agilent ZORBAX Eclipse Plus C18 column (1.8 µm, 2.1 mm × 50 mm) with a ZORBAX Eclipse Plus UHPLC guard column (1.8 µm, 2.1 mm × 5 mm). The mobile phase A, the aqueous phase, was consisted of deionized water with 0.1% formic acid. And the mobile phase B, the organic phase, was prepared by adding 0.1% formic acid in acetonitrile (ACN). The total eluent flow of mobile phase was kept at 0.3 mL/min with gradients changing as follow: 95% A at 0 min, and decreased to 5% until 4 min; increased to 95% in 1 min; then equilibrated for 0.5 min. The total measurement is 5.5 min.

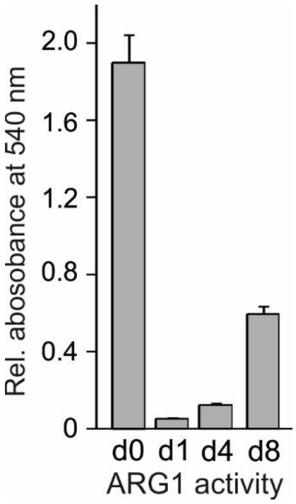

Figure S9. Colorimetric arginase activity assay in HSC lysates. HSC lysates were obtained at day 0, 1, 4 and 8 and used for colorimetric arginase activity assay. The method is described in material and method section using the color change of ISPF in a reaction with urea. Arginase activity was highest at day 0, decreases strongly at day 1 and rises again at day 4 and 8. The error bars indicate S.D. (n=3)

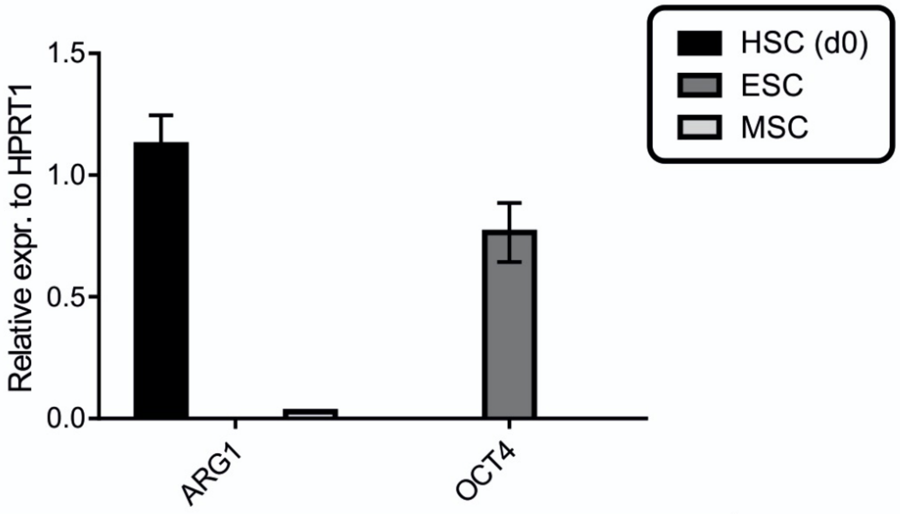

	ARG1			OCT4		
	Mean	SD	N	Mean	SD	N
HSC (d0)	1.124204	0.120954	3	0.000189	0.000052	3
ESC	0.000196	0.000116	3	0.764510	0.121419	3
MSC	0.027531	0.000191	3	0.000002	6.487590e-007	3

Figure S10. qRT-PCR analysis of ARG1 and OCT4 in HSCs, ESCs and MSCs. The mRNA levels of arginase 1 and Oct4 were determined by qRT-PCR measurements in a triplicate. The expression was normalized to the housekeeper Hprt1. HSC, hepatic stellate cell; ESC, embryonic stem cell; MSC, mesenchymal stem cell.
